# Histomorphometric and developmental analysis of human fetal caecum and appendix with its embryological significance

**DOI:** 10.1007/s00276-024-03480-0

**Published:** 2024-09-24

**Authors:** Abhinav Nehra, Chirag Gupta, Vikram Palimar, Sneha Guruprasad Kalthur, Chandni Gupta

**Affiliations:** 1 Kasturba Medical College, Manipal, Manipal Academy of Higher Education, Manipal, 576104 India; 2 Department of Forensic Medicine and Toxicology, Kasturba Medical College, Manipal, Manipal Academy of Higher Education, Manipal, 576104 India; 3 Department of Anatomy, Kasturba Medical College, Manipal, Manipal Academy of Higher Education, Manipal, 576104 India

**Keywords:** Appendix, Caecum, Histomorphometry, Congenital anomalies, Development

## Abstract

**Purpose:**

The variable positions of the appendix can mislead surgeons and physicians to a wrong diagnosis. When appendicitis happens in subhepatic caecum, it can be misdiagnosed and can lead to severe complications during surgical procedures. Therefore, this study aimed to understand the histomorphometric development of the appendix and caecum and to identify when lymphoid follicles appear in the appendix during fetal life.

**Methods:**

The study was conducted on a total of 50 fetuses. The caecum and appendix were carefully dissected. Their position and various measurements were observed. Afterwards, the appendix was taken out for histological processing. All three layers, mucosa, submucosa, and muscularis externa were measured using Image Analyzer Software Image Pro Premiere 9.1, and the appearance of lymphoid follicles was also examined. Results were analyzed using SPSS statistical software.

**Results:**

During the 1st, 2nd, and 3rd trimesters the most common caecum type was type 1: as a lengthy tube, type 3: The lateral wall expanded more, thus it has an asymmetric saccule, and type 4: adult-like caecum. The caecum was mostly situated in the right lumbar region in the 2nd and 3rd trimesters. In the 1st trimester, it was subhepatic in position. The most common position of the appendix was 11 o’clock in 1st and 3rd trimesters. 2nd trimester’s most common position of the appendix was 12 o’clock. The thickness of the mucosa, submucosa, and the muscularis externa increases as the trimester increases. The lymphoid follicles have appeared during the 2nd trimester.

**Conclusion:**

The knowledge from this study will be useful in the diagnosis and treatment of malformations, pathology, and anomalies of the caecum and appendix due to congenital causes.

## Introduction

During development sometimes the caecum and appendix remain below the liver and when such an appendix gets inflamed, it can be misdiagnosed and can lead to severe complications during surgery. While doing surgery if the surgeon fails to identify this malposition it can lead to complications [10].

During embryonic life, the primordium of the cecum and appendix develops in the sixth week as a bud from the antimesenteric margin of the post-arterial segment of the midgut loop [9].

During development the gut projects out of the abdominal cavity due to less space. At around the tenth week, the abdominal cavity grows, and the mid-gut starts returning into the abdominal cavity [2, 12]. The post-arterial segment from which the caecal bud was developing is the last part of the gut to return into the abdominal cavity. While doing so, the appendix develops as a slender diverticulum from the distal end of the caecal bud. When the return is complete the caecum first lies loose near the umbilicus and anterior to the small intestine and superior mesenteric artery. After some time by the straight development of the colon, it carries the caecum upward and towards the right. The colon itself lies across the pedicle of the intestinal mass and superior mesenteric artery while the caecum lies just beneath the liver [11, 14].

For some time by the eleventh week, the caecum and the first portion of the colon lie in the right upper quadrant of the abdomen directly below the right lobe of the liver. Consequently, by obvious retraction of the liver and by straight development of the colon, the caecum attains its final position in the right iliac fossa [9].

During early fetal life, the shape of the caecum is just like a long tube and the appendix develops from the tip of it. Afterward the lateral wall of the caecum develops more rapidly than the medial, thus the attachment site of the appendix comes to lie on the medial side [14]. At 10 weeks of intra-uterine life, after the resolution of the umbilical hernia, the ascending and descending colon adhere to the posterior body wall and their dorsal mesentery slowly fuses permanently with the parietal peritoneum, as this happens the caecum after attaining its position in right iliac fossa around 11th week also becomes fixed [15].

During fetal life the vermiform appendix grows promptly in length, hence at birth, it is a comparatively long, worm-shaped tube that arises from the distal end of the caecum. The positions of the vermiform appendix might vary, and it can be described according to clock hand like retrocaecal (12 o’clock) which is the commonest position, pelvic (4 o’clock), midinguinal (6 o’clock), splenic (pre-ileal, post-ileal 2 o’clock), Promonteric (3 o’clock) paracolic (11 o’clock) [5, 16].

The microscopic features of the appendix comprise 4 coats- Mucosa, submucosa, muscularis externa, and serosa. Crypts are seen. Mucosa has 3 layers- epithelium lined by simple columnar cells, lamina propria, and muscularis mucosa which gets disrupted due to the presence of lymphoid follicles extending into the submucosa. The submucosa contains connective tissue and blood vessels with lymphoid follicles. Muscularis externa comprises of an inner circular and outer longitudinal layer. The serosa is lined by mesothelial cells. During fetal life, lymphoid aggregations were noticed in the lamina propria at about the 20th week of gestation. It increased in number and shape towards the 3rd trimester [4].

While conducting surgeries on appendix its normal development should be known for clinicians, and they can keep in mind some congenital anomalies like subhepatic caecum, malformations of the mobile cecum which occur when the ascending colon mesentery fails to get fixed with the posterior parietal peritoneum etc. These anomalies can lead to cecal volvulus or malposition of the cecum and can occasionally need urgent operation for a situation that might look like acute appendicitis [17, 18]. The inverse cecum sometimes gets fixed in the subhepatic region, and the upcoming elongation of the ascending colon pushes it to curve cranially [19]. Therefore, this study was conducted to get detailed information on the morphometric and histological development of the caecum and appendix.

## Materials and methods

Study type: Observational study.

Sample size: The research was conducted on 50 aborted human fetuses. The gestational age of fetuses was identified by measuring their Crown- rump and Crown- heel length. The total number of fetuses was categorized into 3 gestational age groups-.

Group 1 (1st Trimester): Gestational age is < 12 weeks (10 fetuses- 4 females and 6 males).

Group 2 (2nd Trimester): Gestational age 13–24 weeks (20 fetuses- 10 males and females each).

Group 3 (3rd Trimester): Gestational age > 24 weeks (20 fetuses- 10 males and females each).

Institutional Ethics Committee clearance was obtained before starting the study. (IEC: 203/2020).

Study period: April 2022 to September 2023.

### Inclusion criteria

Fetuses without any gross external congenital anomalies were included in the study.

### Exclusion criteria

Fetuses aborted due to birth defects.

Instruments used: The materials used for the various measurements included Vernier calipers, a microscope with image analyzer software Image Pro Premier 9.1.

### Procedure

**Fig. 1:**
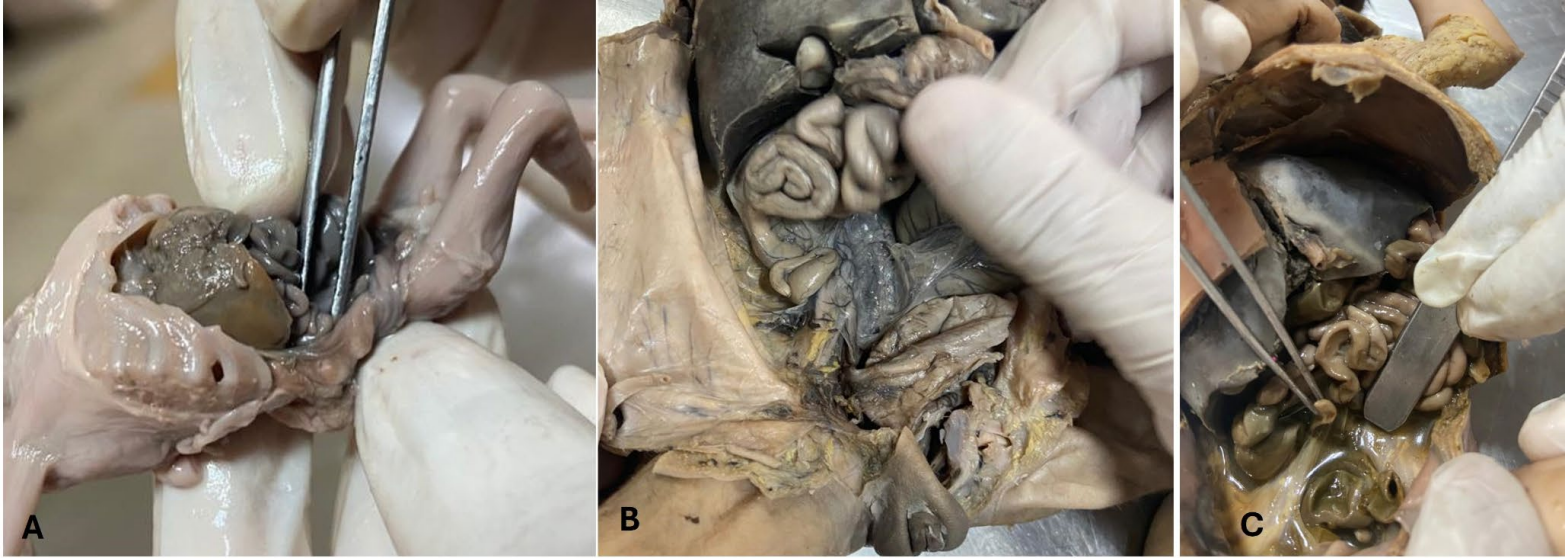
Various positions of caecum. (**A**) Subhepatic, (**B**) Right lumbar, (**C**) Right iliac

**Fig. 2:**
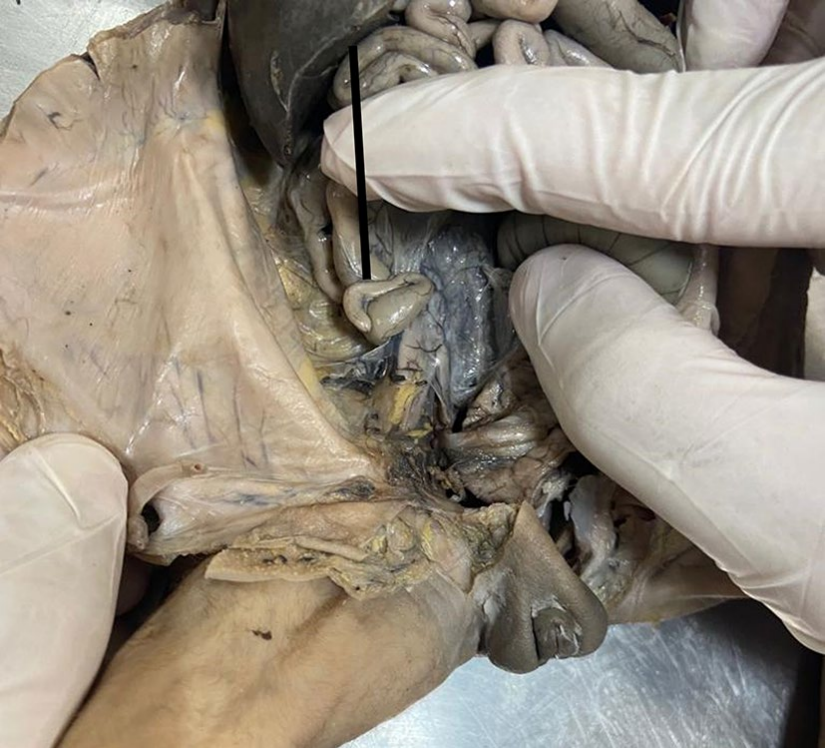
Measurement of distance of caecum from the inferior border of liver when caecum was not subhepatic in position

**Fig. 3:**
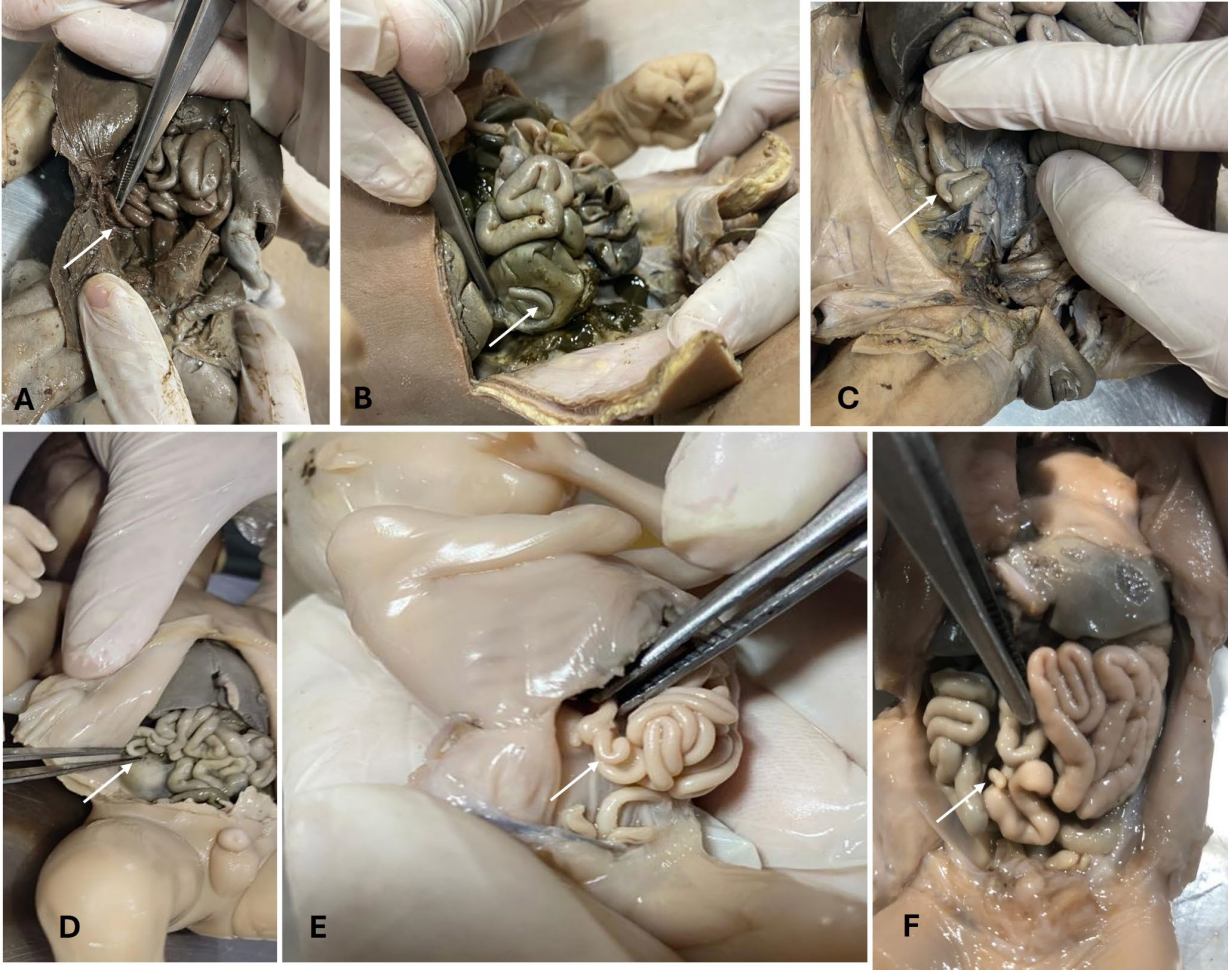
Various positions of appendix. **A** 11o’clock, **B** 12o’clock, **C** 6o’clock, **D** 2o’clock, **E** 3o’clock **F** 4o’clock

The Foetus abdomen was dissected. The position of the caecum and appendix (Subhepatic, right lumbar, and right iliac) was noted (Fig. 1). If it was not below the liver its distance from the liver was noted (Fig. 2). Position of appendix retrocaecal (12 o’clock), paracolic (11 o’clock), splenic (2 o’clock), promonteric (3 o’clock), pelvic (4 o’clock), and midinguinal (6 o’clock) was noted (Fig. 3). Its length and diameter were observed. The length of the mesoappendix was also observed. All measurements were taken thrice by the same person to increase the accuracy of measurement.

The caecum was divided into various types as described previously: [14]

Type 1: Caecum has a lengthy tube.
Type 2: Tube-like caecum with both sides expanded equally on both sides and appendix located at its tip.
Type 3: The lateral wall expanded more, therefore it has an asymmetric saccule.
Type 4: Adult-like caecum with extremely expanded lateral wall as compared to medial.
Type 5: Atypical caecum.

**Fig. 4:**
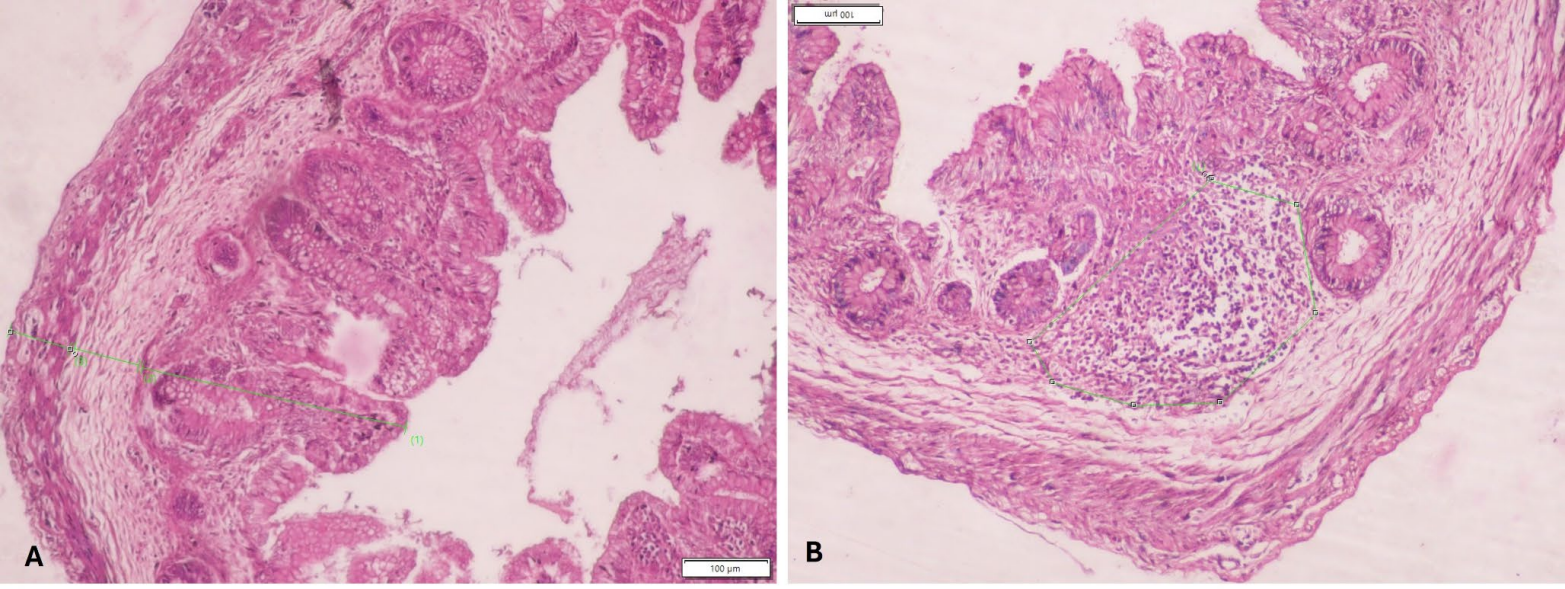
H&E-stained microscopic images of appendix during 2nd trimester at 10x magnification. **A** Measurement of various layers of appendix. **B** Measurement of area of lymphatic follicle

For histological processing, the appendix samples were sliced up. Initially, they were fixed in a 10% formalin solution. Then the tissues were dehydrated using ascending grades of alcohol, i.e. in 50% and 70% alcohol for 1 day each followed by 90% alcohol for 8h, and finally in 100% alcohol for 6–8h. The tissues were then submerged in xylol for clearing till the tissue became transparent. Subsequently, they were kept for impregnation in molten paraffin wax containers for 2h. The paraffin embedding was done according to the procedure of Carleton [3]. Successive sections were taken, and later tissues were mounted and fixed on the glass slides using standard technique. The slides were then stained with Hematoxylin and Eosin (H&E). The microscopic features were observed utilizing a light microscope. The image analyzer software Image Pro Premier 9.1 was used to measure the histomorphometry of the appendix and even for capturing the images. The thickness of all the 3 histological layers was measured. The appearance of lymphoid follicles was also observed. The total area occupied by them was also measured (Fig. 4).

Statistical parameters like mean and standard deviation were calculated for each group. Independent T-Test was done to compare between males and females for each parameter within each trimester. A two-way ANOVA test was done to compare parameters between each group followed by Tukey’s post hoc test, was done to compare parameters within each group using SPSS 20. Software.

## Results

### Gross parameters

Measurements done related to caecum and appendix in each group are shown in Table 1.

**Table 1:** Showing measurements done related to caecum and appendix in each trimester

Trimesters		Average distance from liver (mm)	Length of appendix (mm)	Diameter of appendix (mm)	Length of mesoappendix (mm)
1st	Male	2.08 ± 2.83	6.33 ± 1.86	0.16 ± 0.16	3.16 ± 1.60
	Female	3.25 ± 1.70	8.50 ± 1.73	0.12 ± 0.05	4.00 ± 2.16
2nd	Male	11.70 ± 6.03	16.45 ± 3.73	1.24 ± 0.54	6.51 ± 2.16
	Female	9.55 ± 11.26	21.15 ± 5.93	1.53 ± 0.77	5.91 ± 3.22
3rd	Male	15.65 ± 12.61	24.7 ± 10.40	1.6 ± 1.29	7.85 ± 6.16
	Female	19.15 ± 10.50	24.8 ± 8.09	2.4 ± 0.67	7.35 ± 5.15

When all parameters were compared in the 3rd group between genders there was no significant correlation seen as p values were 0.509, 0.981, 0.09, and 0.846. for distance from the liver, length of appendix, diameter of appendix, and length of mesoappendix.

When all parameters were compared in the 2nd group between genders there was no significant correlation seen in the distance from the liver, the diameter of appendix, and length of mesoappendix as the p values were 0.601, 0.34, and 0.863. While significant correlation was seen in the length of appendix as the p value was 0.04.

When all parameters were compared in the 1st group between genders there was no significant correlation seen as p-values were 0.441, 0.101, 0.579, and 0.501 for distance from the liver, length of the appendix, the diameter of appendix, and length of mesoappendix.

When comparing parameters between the groups by Anova test there was a significant correlation seen as p-value was 0.001, 0.000, 0.000, 0.037 for distance from the liver, length of the appendix, the diameter of appendix, and length of mesoappendix.

When comparing parameters within the groups by Post hoc Tukey’s test there was a significant correlation seen for distance from the liver within groups 1 and 3 as p-value was 0.000. The length of the appendix was significantly correlated within all the groups as p-value was < 0.05. The diameter of appendix was significantly correlated within groups 1 and 2 as the p-value was 0.001 and groups 1 and 3 it was 0.000. The length of the mesoappendix was significantly correlated within groups 1 and 3 as p value was 0.028 (Table 2).

**Table 2:** Comparing parameters within groups using post hoc Tukey’s test

Parameters	Group	Groups	P value
Distance from liver	1	2	0.073
		3	0.000*
	2	1	0.073
		3	0.064
	3	1	0.000*
		2	0.064
Length of appendix	1	2	0.000*
		3	0.000*
	2	1	0.000*
		3	0.021*
	3	1	0.000*
		2	0.021*
Diameter of appendix	1	2	0.001*
		3	0.000*
	2	1	0.001*
		3	0.053
	3	1	0.000*
		2	0.053
Length of mesoappendix	1	2	0.200
		3	0.028*
	2	1	0.200
		3	0.503
	3	1	0.028*
		2	0.503

*P value < 0.05 was considered as significant

### 3rd trimester (Group 3)

In the female’s caecum and appendix were in the right lumbar, right iliac fossa, and subhepatic in 50%, 30%, and 20% of cases. In male’s caecum and appendix were in the right lumbar and right iliac fossa in 40% and 60% of cases.

In the females and males’, the position of appendix was retrocaecal (12o, clock), paracolic (11 o’clock), inguinal (6 o’clock), splenic (2 o’clock), and pelvic (4 o’clock) in 20% each, 30% each, 30% and 10%, 10% and 30%, 10% in each of cases. There was no promonteric position observed.

In females, all caecum (100%) belongs to type 4 while in males 40% belongs to type 3 and 60% belongs to type 4.

### 2nd trimester (Group 2)

In the female’s caecum and appendix were in the right lumbar, right iliac fossa, and subhepatic in 40%, 10%, and 50% of cases. In male’s the caecum and appendix were in the right lumbar and subhepatic in 70% and 30% of cases.

In the females and males’, the position of the appendix was retrocaecal (12o, clock), paracolic (11 o’clock), inguinal (6 o’clock), promonteric (3 o’clock), and pelvic (4 o’clock) in 30% each, 20% each, 10% and 20%, 20% and 10% and 20% in each of cases. There was no pelvic position observed.

In females 10% of the caecum belongs to type 2, 70% belongs to type 3 and 20% belongs to type 4 while in males 20% of the caecum belongs to type 2, 70% belongs to type 3, and 10% belongs to type 4.

### 1st trimester (Group 1)

In the female’s caecum and appendix were in 50% of cases each in the right lumbar and subhepatic region. In male’s caecum and appendix were in the right lumbar and subhepatic in 33.33% and 66.67% of cases.

In the female’s position of the appendix was retrocaecal (12o, clock), inguinal (6 o’clock), splenic (2 o’clock), and pelvic (4 o’clock) in 25% of cases each. There was no paracolic and promonteric position observed in females. In the male position of the appendix was retrocaecal (12 o, clock), paracolic (11o’clock) and inguinal (6 o’clock) in 16.67%, 50%, and 33.33% of cases. There was no splenic, promonteric, and pelvic position observed in males.

In females 50% of caecum each belongs to type 1 and 2 while in males all caecum (100%) belongs to type 1.

### Histology parameters

The thickness of all histological layers of the appendix in all trimesters of the fetus is shown in Table 3.

**Table 3:** Showing the thickness of all histological layers of appendix

Trimester	Mucosa	Submucosa	Muscularis externa
1st	110.73 ± 12.16	52.13 ± 8.72	39.53 ± 7.34
2nd	220.44 ± 58.97	75.11 ± 24.59	81.98 ± 16.47
3rd	187.8 ± 52.22	88.99 ± 41.56	100.38 ± 36.29

### Mucosa

**Fig. 5:**
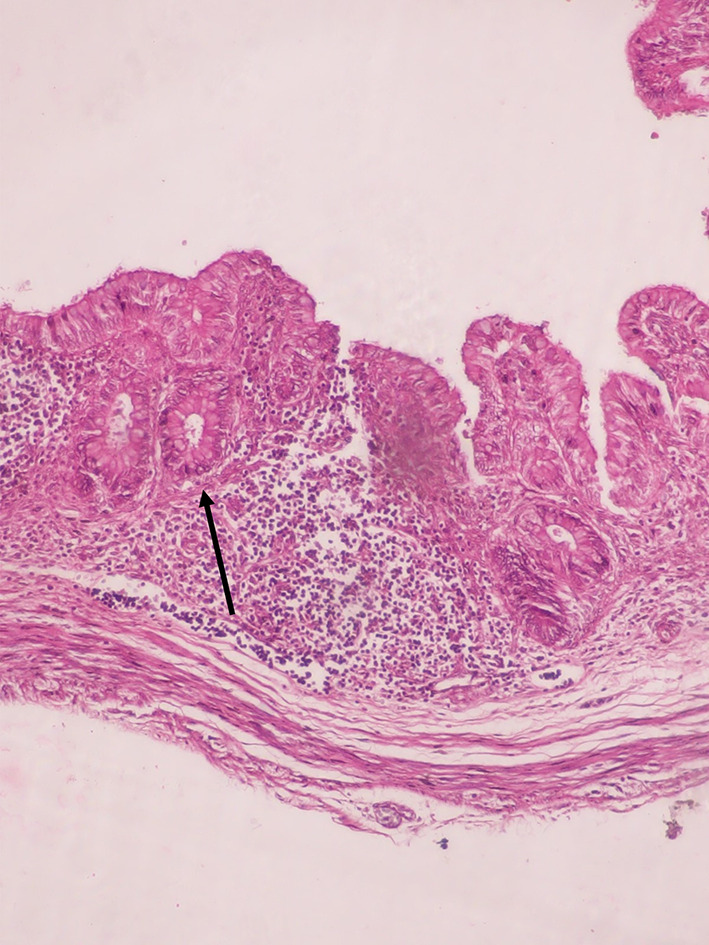
H&E-stained microscopic images of appendix during 2nd trimester at 10x magnification with appearance of muscularis mucosa

The epithelium was simple columnar with well-developed goblet cells. Crypts and intestinal glands were also developed in the lamina propria during the 1st trimester. Lymphatic follicles were scattered and diffused in the lamina propria during the 2nd trimester. Muscularis mucosa was well-developed in the 2nd trimester (Fig. 5).

Appendix mucosa appeared to be thinner in the first trimester. During the second trimester, the width of the mucosa increased. In the third trimester, the mucosa appears to be thinner than in the second trimester but thicker than in the first trimester. The width of mucosa increased both due to the development of epithelium and the increase in the width of lamina propria.

While correlating mucosa development during the three trimesters it was noted that a significant correlation was noted between1st and 2nd trimesters as (P value=0.047) and between 1st and 3rd trimesters as (P value=0.010) while between 2nd and 3rd trimesters it was not significant (Table 4).

**Table 4:** Showing comparisons drawn for mucosa, submucosa and muscularis externa amongst the three trimesters

Trimester	Mucosa	Submucosa	Muscularis externa
1st and 2nd	0.047*	0.289	0.004*
1st and 3rd	0.010*	0.048*	0.049*
2nd and 3rd	0.462	0.716	0.413

*P value is considered significant < 0.05 level (2-tailed)

### Submucosa

The width of the submucosa in the appendix goes on increasing as the increase in trimesters. Submucosal plexus was seen during the first trimester. During 2nd and 3rd trimester the area occupied by the plexus increases due to increase in the cell proliferation. In submucosa, lymphatic follicles were seen after disrupting the muscularis mucosa. Connective tissue and blood vessels in the submucosa were also appreciated.

While correlating submucosa development during the three trimesters it was noted that a significant correlation was noted between 1st and 3rd trimesters as (*P* value = 0.048) while between 1st and 2nd trimesters and between 2nd and 3rd trimesters it was not significant (Table 4).

### Muscularis externa

The width of the muscularis externa in the appendix goes on increasing as the increase in trimesters. Both circular and longitudinal muscles were seen to be well-developed in the 1st trimester (Fig. 4). Myenteric plexus was seen during the first trimester. During the 2nd and 3rd trimester the area occupied by the plexus increases due to increase in the cell proliferation.

While correlating muscularis externa development during the three trimesters it was noted that a significant correlation was observed between 1st and 2nd trimesters as (*P* value=0.004) and between 1st and 3rd trimesters as (*P* value=0.049) while between 2nd and 3rd trimesters it was not significant (Table 4).

### Appearance of lymphatic follicles

Lymphatic follicles were scattered and diffused in the lamina propria during the 2nd trimester. During the 3rd trimester, they got organized and lymphatic nodules with germinal centers were well-developed. The area occupied by them was 50,349mm2 during the 2nd trimester and later it became 35,266mm2. This may be due to the good organization of the lymphatic follicles with the germinal centers (Fig. 4).

**Fig. 6:**
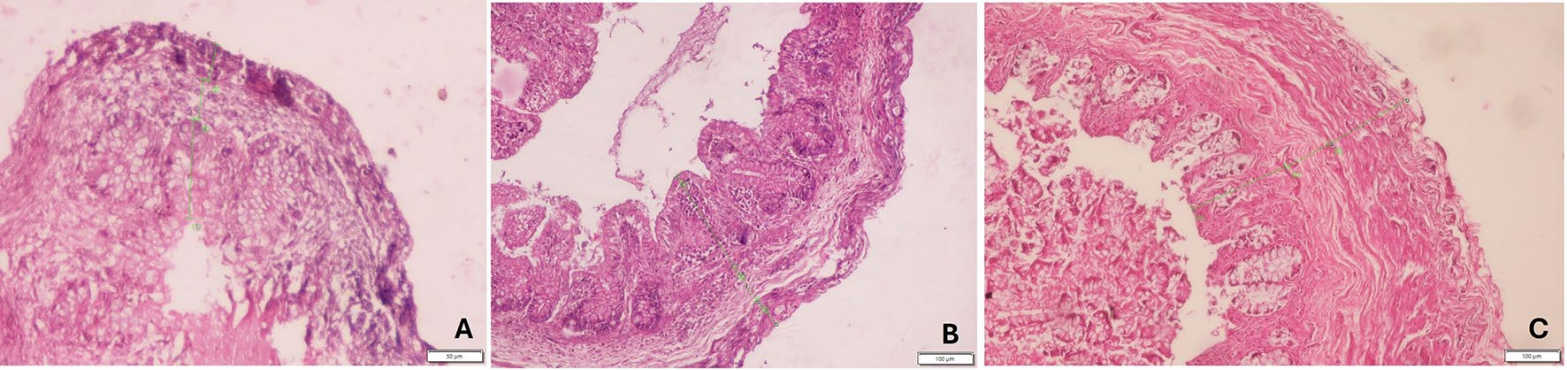
H&E-stained microscopic images of appendix at 10x magnification. **A** 1st trimester, **B** 2nd trimester, **C** 3rd trimester

The histological comparison of all the trimesters in the picture is shown in Fig. 6.

## Discussion

The most common parts of the large intestine that can have maximum topographical variation or are characterized by changes in location and morphology are the caecum and appendix. That’s why normal gross and histological development of both is very important to have a better knowledge of their different positions and other morphological features [10].

A comparison of gross features of the caecum and appendix between different authors and the present study is shown in Table 5.

**Table 5:** Showing comparison of gross features of caecum and appendix between different authors and the present study

Authors	Position of caecum	Shape of caecum	Position of appendix	Length of vermiform appendix	Length of mesoappendix
Nidhi et al. [10]	Subhepatic caecum in18 foetuses, right lumber in 15 foetuses and in right iliac fossa in 5 foetuses	Conical in 19 foetuses, quadrangular in 10 foetuses and in 9 foetuses’ caecum is right saccular			
Malasa et al. [6]		Conical caecum was seen mainly during the foetal period. An asymmetrically shaped saccule was noticed during the later stages of the foetal life	2o’clock position of appendix was seen mainly in female foetuses (34%), and a 11o’clock (48%) position was noticed mostly in male foetuses.	1st, 2nd, 3rd trimester was 6,16, 29 mm	1st, 2nd, 3rd trimester was 5,8,13 mm
Present study	Subhepatic caecum was observed in 15 foetuses, 23 were right lumbar and 12 were right iliac.	37 were right saccular, 8 were conical and 5 were quadrangular.	12o’clock position of appendix was most common in female foetuses (24%), while in males 11o’clock position was most common (32%).	1st, 2nd, 3rd trimester was 7.40,18.80, 24.75 mm and	1st, 2nd, 3rd trimester was 3.58,6.21,7.60 mm.

Mohammad et al conducted a study on 25 vermiform appendix samples from the fetuses of 17–40 weeks gestational ages. They found that at the 17th week crypts in mucosa were noticed. Intestinal glands and diffuse lymphocytes were seen in lamina propria. The lining epithelium was columnar with a striated border and the goblet cell population was fewer. Muscular coats were well-defined. In the 20th week lymphoid follicles were noticed in the mucosa and submucosa. At the 29th week dense aggregation of lymphocytes was seen in lamina propria. At the 34th and 39th weeks clear muscularis mucosae might be recognized [8]. In the present study during the 1st trimester crypts, intestinal glands were seen, lining epithelium was columnar, the goblet cell population was noticed, and muscular coats were also well defined. During the 2nd trimester, lymphoid follicles were observed but they were diffuse in mucosa and submucosa and clear muscularis mucosae could be identified. During the 3rd trimester dense aggregation of lymphocytes was noticed in lamina propria.

Malasa et al conducted a study on 80 human fetuses of gestational ages between 10–40 weeks of gestation. They found that the thickness of serosa, muscularis externa, and mucosa increased with gestational age. The lymphocyte aggregation was initially noticed in the 17th week of the fetal period. While comparing the thickness of mucosa there were no differences between second, third trimester, and full-term fetuses (*p* > 0.05), while a significant difference was noticed in the thickness of serosa and muscularis externa between the various trimesters (*p* value > 0.05) [7]. In the present study, mucosa appeared to be thinner in the first trimester. During the second trimester, the width of the mucosa increased. In the third trimester, the mucosa appears to be thinner than second trimester but thicker than in the first trimester. While the width of the submucosa and muscularis externa goes on increasing with respect to the trimester. The lymphocyte aggregation was initially noticed during the 2nd trimester of the fetal period. While correlating mucosa development during the three trimesters it was observed that a significant correlation was there between 1st and 2nd trimesters (*P* value = 0.047) and between 1st and 3rd trimesters (*P* value = 0.010) while between 2nd and 3rd trimesters it was not significant. While correlating submucosa development during the three trimesters it was observed that a significant correlation was there between 1st and 3rd trimesters (*P* value = 0.048) while between 1st and 2nd trimesters and between 2nd and 3rd trimesters it was not significant. While correlating muscularis externa development during the three trimesters it was observed that a significant correlation was there between 1st and 2nd trimesters (*P* value = 0.004) and between 1st and 3rd trimesters (*P* value = 0.049) while between 2nd and 3rd trimesters it was not significant.

In the present study, the position of the caecum was subhepatic in 15 fetuses, 23 were right lumbar and 12 were right iliac. This shows that the position of the caecum while development shifted and can vary which can lead to complications if not known while conducting surgeries. The location of the appendix changed according to the caecum type during the fetal period. The position of the appendix was also observed. The most common position was the 12o’clock position in female fetuses (24%), while in males 11 o’clock position was the most common (32%). This is also important to note as splenic pre-ileal is a dangerous position because if it gets inflamed infection can spread to the whole abdomen. The length of the mesoappendix increases as the trimester increases. Ajmani and Ajmani and Solanke stated that the attachment length of the meso-appendix could be significant in clinical appendicitis since the features of the mesoappendix are important in the evaluation of appendicitis cases [1, 13].

Subhepatic caecum and appendix, when inflamed, might lead to misdiagnosis and severe complications during surgeries. Failure to recognize this malposition can lead to dangerous blunders in procedure. Hence, Surgeons should have the proper awareness and knowledge of the development of the caecum and appendix which will help them to know any variations that might occur in adult life.

## Conclusion

The study was conducted on a total of 50 fetuses. The caecum and appendix were dissected carefully. Their position and various measurements were noted down. Later the appendix was taken out for histological processing. All 3 layers, mucosa, submucosa, muscularis externa were measured and the appearance of lymphoid follicles was also observed. During the 1st, 2nd, and 3rd trimesters the most common caecum type 1, 3 4. The caecum was mostly situated in the right lumbar region in the 2nd and 3rd trimesters. In the 1st trimester, it was subhepatic in position. Most frequent position of the appendix was 11 o’clock in 1st and 3rd trimesters. 2nd trimester’s most frequent position of the appendix was 12 o’clock. The thickness of the submucosa and the muscularis externa increases as the trimester increases. The appearance of the lymphoid follicles in the 2nd trimester of the appendix was observed under a microscope.

## Limitations of the study

1. The number of fetuses collected in this study for the 1st trimester was less as compared to 2nd and 3rd trimester.
2. Fetuses in the present study were divided into three groups- 1st, 2nd and 3rd trimester and the data were collected according to these groups. Hence, it is difficult to present specific age in weeks when the structures appear in microscopic findings.

## Data Availability

No datasets were generated or analysed during the current study.
